# Cerebral infarction in advanced non-small cell lung cancer: a case control study

**DOI:** 10.1186/s12885-016-2233-1

**Published:** 2016-03-10

**Authors:** Motoyasu Kato, Takehito Shukuya, Keita Mori, Ryota Kanemaru, Yuichiro Honma, Yuta Nanjo, Keiko Muraki, Rina Shibayama, Ryo Koyama, Naoko Shimada, Fumiyuki Takahashi, Kazuhisa Takahashi

**Affiliations:** Department of Respiratory Medicine, Juntendo University Graduate School of Medicine, 3-1-3, Hongo, Bunkyo-ku, Tokyo, 113-8431 Japan; Clinical Trial Coordination Office, Shizuoka Cancer Center, 1007 Shimonagakubo, Nagaizumi-cho, Suntou-gun, Shizuoka 411-8777 Japan

**Keywords:** Non-small cell lung cancer, Trousseau syndrome, Thrombosis, Cerebral infarction, Brain metastasis

## Abstract

**Background:**

Advanced non-small cell lung cancer (NSCLC) patients often develop thromboembolic events, including cerebral infarction (CI). However, the relationship between advanced NSCLC and CI has not been thoroughly investigated. We examined the association between advanced NSCLC and CI and risk factors for CI in advanced or post-operative recurrent NSCLC patients.

**Methods:**

We retrospectively investigated 515 patients diagnosed with advanced or post-operative recurrent NSCLC at Juntendo University Hospital between April 2009 and March 2014.

**Results:**

Among the 515 patients evaluated, 15 patients (2.9 %) developed CI after diagnosis of advanced or post-operative recurrent NSCLC. Univariate and multivariate analyses were conducted, and brain metastasis was the only significant independent risk factor for CI (odds ratio 5.24, 95 % confidence interval 1.72–16.10, *p* = 0.004). The incidence was 6.3 % in these patients. The median survival time was 36 days, and 1-year survival rate was 6.7 % after development of CI. Overall survival from diagnosis of advanced NSCLC or post-operative recurrence was significantly shorter in patients with CI than in patients without CI (223 days versus 895 days; HR, 3.46; 95 % confidence interval, 2.04–6.02; *p* = 0.001).

**Conclusions:**

The incidence of CI is high in advanced or post-operative recurrent NSCLC, and is especially higher in patients with brain metastasis than in those without brain metastasis. Moreover, CI may affect patient’s prognosis. Careful monitoring for the development of CI in patients with advanced or post-operative recurrent NSCLC is needed, especially for patients with brain metastasis.

## Background

Cancer is the leading cause of death in the world. Non-small cell lung cancer (NSCLC) is one of the most aggressive diseases and has a poor prognosis compared with other malignancies. NSCLC patients are usually diagnosed at an advanced stage and usually receive chemotherapy. The prognosis of advanced NSCLC patients is improving due to the developments in chemotherapy, and better control of adverse events and complications is becoming more important.

An association between cancer and thrombotic events was first reported by Trousseau in 1865 [1]. The association between cancer and thrombotic disease including cerebral infarction (CI) is referred to as Trousseau syndrome. Patients with solid tumors, including lung, breast, ovary and pancreas cancers, have significantly higher risk of thromboembolic complications than patients with hematologic malignancies such as leukemia and malignant lymphoma [2]. In another report, the prevalence of cancer was higher in stroke patients than in the general population (*p* = 0.001). The most common cancer types were colorectal cancer (20.2 %), prostate cancer (15.6 %), breast cancer (12.7 %), cancer of the urinary tract system (10.3 %), gynecological cancer (6.2 %) and lung cancer (4.5 %) [3].

CI impairs activities of daily living and performance status (PS). Most patients with CI will not be able to continue anticancer treatment. Therefore, the survival of cancer patients with CI is poor.

Although some previous papers reported the incidence of CI in lung cancer patients, the incidence rate was based on data from a registry including all lung cancer patients, from early stage to advanced stage [4, 5]. Moreover, the effect of CI on the prognosis of advanced NSCLC patients has not been reported. The aim of this study was to investigate the association between CI and advanced or post-operative recurrent NSCLC, risk factors for CI in advanced NSCLC, and the effect of CI on the prognosis of advanced NSCLC.

## Methods

### Study design

Between April 2009 and March 2014, 532 patients were diagnosed with unresectable stage IIIA, IIIB, or IV or post-operative recurrent NSCLC at the Juntendo University Hospital. Seventeen patients were transferred to other hospitals immediately after diagnosis. A total of 515 patients attended our hospital and were enrolled in this case control study. We divided all patients into two groups, patients with and without CI. Then, we evaluated the differences between the two groups in any items.

Data regarding the patients’ baseline characteristics at the time of diagnosis of advanced NSCLC or post-operative recurrence were retrospectively collected, including age, sex, smoking history, Eastern Cooperative Oncology Group (ECOG)-PS, histological type, clinical stage, brain metastasis, and complications (hypertension, diabetes, hyperlipidemia, atrial fibrillation and old myocardial infarction).

CI was firstly detected by symptom and neurological examination and confirmed by brain magnetic resonance imaging (MRI) including diffusion-weighted MRI and neurologist. Blood examinations, especially d-dimer levels, were evaluated in patients with CI. For patients with CI, the clinical course before and after CI and survival time from the onset of CI were evaluated. Overall survival (OS) from diagnosis of advanced NSCLC or post-operative recurrence after radical surgery or chemoradio/radiotherapy was evaluated and compared between patients with CI and without CI.

All patients involved in this study provided verbal informed consent for the use of their medical data. This study protocol was approved by the Juntendo University Ethical Committee and registered under number 26–635.

### Statistical method

We used the chi square test, Fisher's exact test, or Wilcoxon two-sample test to compare patient characteristics. Univariate and multivariate analyses were performed using logistic regression to assess the risk factors for CI. OS was plotted using the Kaplan–Meier method. Differences in OS were analyzed using the log-rank test, and hazard ratio (HR) was calculated by cox proportional hazard model. All *p-*values <0.05 were considered statistically significant. All statistical analyses were performed using SPSS ver. 15.0 for Windows (Chicago, IL, USA).

## Results

### Incidence of CI and patient characteristics

**Fig. 1:**
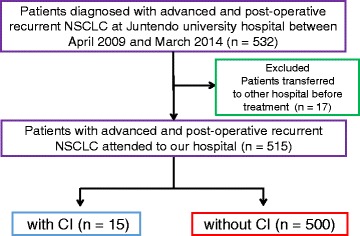
Patients selection

Between April 2009 and March 2014, 532 patients were diagnosed with advanced or post-operative recurrent NSCLC. Seventeen patients were excluded from this research because they were transferred to other hospitals immediately after diagnosis. Thereafter, 515 patients were enrolled in this case–control study (Fig. 1). Fifteen out of 515 patients (2.9 %) developed CI after being diagnosed with advanced NSCLC or post-operative recurrence after radical surgery or chemoradio/radiotherapy. The baseline characteristics of patients with and without CI are listed in Table 1. There were no significant differences in age, sex, smoking history, ECOG-PS, disease stage and tumor histology between the two categories. Additionally, there were no significant differences in hypertension, diabetes, hyperlipidemia, atrial fibrillation, and old myocardial infarction between patients with and without CI. More male patients developed CI, although there was no significant difference (*p =* 0.11).

**Table 1:** Patients characteristics

		Without CI *N* = 500	With CI *N* = 15	*P*
Age (year)	Median (range)	66 (34–92)	64 (47–78)	0.39
Sex	Male (%)	293 (58.6 %)	12 (80.0 %)	0.11
Smoking history	Yes (%)	316 (63.2 %)	12 (80.0 %)	0.19
PS	0,1 (%)	484 (96.8 %)	14 (93.3 %)	0.42
Histology	Adenocarcinoma (%)	411 (82.2 %)	12 (80.0 %)	0.66
	Advanced (%)	381 (76.2 %)	12 (80.0 %)	0.97
	Post-operative recurrence (%)	119 (23.8 %)	3 (20.0 %)	
Hypertension	Yes (%)	213 (42.6 %)	7 (46.7 %)	0.75
Diabetes	Yes (%)	93 (18.1 %)	2 (13.3 %)	0.61
Old myocardial infarction	Yes (%)	46 (9.2 %)	2 (13.3 %)	0.77
Hyperlipidemia	Yes (%)	95 (18.7 %)	1 (6.7 %)	0.25
Atrial fibrillation	Yes (%)	23 (4.5 %)	1 (6.7 %)	0.71

*CI* cerebral infarction, *PS* performance status

### Cerebral infarction and risk factors

Patients who developed CI are listed in Table 2. Thirteen patients was diagnosed with advanced NSCLC and 2 diagnosed with post-operative recurrence NSCLC. CI was first detected by symptom and neurological examination, and confirmed by a neurologist with brain MRI finding. All patients had some symptoms at the onset of CI. Twelve patients had hemiplegia, 10 had dysarthria, and 5 had disturbance of consciousness. Ten patients had multiple CIs and 5 patients had a single CI on brain MRI. Fourteen patients developed CI during anticancer therapy, including radiation therapy (2 patients) and chemotherapy (12 patients). One patient was not able to receive anticancer treatment because of advanced age and dementia. In two patients who developed CI during radiation therapy, one patient received only radiation therapy before onset of CI, and the other patient had been treated with carboplatin (CBDCA), pemetrexed (PEM) plus epidermal growth factor receptor (EGFR) tyrosine kinase inhibitor (TKI), gefitinib followed by radiation therapy before onset of CI. Seven patients developed CI during first line chemotherapy. Of these patients, 2 patients treated with CBDCA plus paclitaxel (PTX), 2 received cisplatin (CDDP) plus PEM, and 1 was treated with CBDCA plus PEM. There was no patient treated with bevacizumab. Four patients developed CI during second line chemotherapy. One patient was treated with docetaxel monotherapy and the other received PEM. Three patients with sensitive mutation of EGFR and 1 with EML4/ALK fusion gene developed CI during treatment with EGFR-TKI, erlotinib, and EML4/ALK inhibitor, crizotinib, respectively. Only 1 patient treated with erlotinib for first line treatment, and the other 2 patients treated for second line. One patient received crizotinib for first line treatment. Eleven patients had progressive disease for anticancer therapy at the onset of CI. Carotid arteries were evaluated by ultrasonography in all patients, and there was no evidence of thromboembolism. Although 1 patient with CI had atrial fibrillation, he received the anti-arrhythmic drug digitalis and the anticoagulant warfarin before the onset of CI. Serum d-dimer level was also high in all patients at the onset of CI (average: 28.1, range: 1.8–141.5 μg/ml). Fourteen out of the 15 patients had to discontinue anticancer therapy.

**Table 2:** Our cerebral infarction cases

	Age ranges	Stage	Histology	Brain metastasis at diagnosis	Number of cerebral infarctions	Anticancer therapy	Chemotherapeutic regimen	D-dimer	Outcome	OS from CI (days)
1	70s	IV	Ad	–	Multi	+	Erlotinib	2.2	Recover	791
2	50s	rec	Ad	–	Multi	+	DTX	5.6	Recover	341
3	60s	IV	Ad	+	Multi	+	CBDCA + PTX	4.9	Recover	280
4	60s	IIIB	Sq	–	Multi	+	CBDCA + PTX	4.6	Recover	161
5	70s	IV	Ad	+	Multi	+	Erlotinib	24.7	Recover	125
6	60s	IV	Sq	+	Mono	+	RTx	1.8	Dead	66
7	70s	IV	Ad	+	Multi	–	–	37.8	Recover	44
8	40s	IV	Ad	+	Multi	+	Erlotinib	10	Dead	36
9	50s	IV	Ad	+	Multi	+	Crizotinib	46	Dead	32
10	60s	IV	La	+	Multi	+	DTX	46.6	Dead	28
11	50s	IV	Ad	+	Mono	+	CDDP + PEM	2.8	Dead	25
12	60s	rec	Ad	–	Mono	+	CBDCA + PTX	60	Recover	17
13	70s	IV	Ad	+	Multi	+	RTx	22.3	Dead	7
14	70s	IV	Ad	–	Mono	+	PEM	141	Dead	1
15	50s	rec	Ad	+	Mono	+	CDDP + PEM	11.3	Dead	1

*rec* recurrence, *Ad adenocarcinoma, Sq squamous cell carcinoma, DTX* docetaxel, *CBDCA* carboplatin, *PTX* paclitaxel, *CDDP* cisplatin, *PEM* pemetrexed, *RTx* radiation therapy

The results of univariate and multivariate analyses to identify risk factors for CI are shown in Tables 3 and 4. Brain metastasis at diagnosis of advanced or post-operative recurrent NSCLC was significantly associated with CI on univariate analysis (odds rate [OR], 4.67; 95 % CI, 1.57–13.89; *p =* 0.006). Multivariate analysis was performed using eleven variables (age, sex, smoking history, ECOG-PS, histology, hypertension, diabetes, hyperlipidemia, atrial fibrillation, old myocardial infarction and brain metastasis at diagnosis) and revealed that only brain metastasis at diagnosis (OR, 5.24; 95 % CI, 1.72–16.10; *p =* 0.004) was a significant independent risk factor for CI. The incidence of CI in patients with brain metastasis was significantly higher than that in patients without brain metastasis (6.3 vs. 1.4 %; *p =* 0.006). The fatality rate was 60 % in patients with brain metastasis, and 40 % in patients without brain metastasis; this difference was not statistically significant (Table 5). Among the patients who developed symptomatic CI, 10 had multiple infarctions. Among the patients with brain metastasis at diagnosis of advanced or postoperative recurrent NSCLC, 7 had multiple micro CIs and 3 had a single large CI. No statistically significant differences were observed between brain metastasis and type of infarction.

**Table 3:** Univariate analysis of risk factors associated with cerebral infarction

	Overall	without CI	with CI	OR	95 % CI	*P*
Overall	515	500	15			
Age				2.04	0.56–7.27	0.39
≦70	344	332	12			
≧71	171	168	3			
Sex				2.33	0.78–10.13	0.11
Female	210	207	3			
Male	305	293	12			
Smoking history				2.32	0.65–8.36	0.19
No	187	184	3			
Yes	328	316	12			
PS				2.16	0.26–17.48	0.42
0–1	498	484	14			
2–3	17	16	1			
Histology				0.71	0.16–3.20	0.66
Ad	423	411	12			
Not Ad	92	89	3			
Hypertension				1.18	0.42–3.32	0.75
No	295	287	8			
Yes	220	213	7			
Diabetes				0.67	0.15–3.03	0.61
No	420	407	13			
Yes	95	93	2			
Hyperlipidemia				0.31	0.04–2.34	0.25
No	419	405	14			
Yes	96	95	1			
OMI				1.49	0.33–6.78	0.77
No	467	454	13			
Yes	48	46	2			
Atrial fibrillation				1.48	0.18–11.75	0.71
No	491	477	14			
Yes	24	23	1			
Brain metastasis				4.67	1.57–13.89	0.006
No	355	350	5			
Yes	160	150	10			

*PS* performance status, *Ad* adenocarcinoma, *OMI* old myocardial infarction, *95 % CI* 95 % confidential interval

**Table 4:** Multivariate analysis of risk factors associated with cerebral infarction

	Odds ratio	95 % CI	*P*
Variable			
Age (≦70 vs ≧71)	1.04	0.30–3.53	0.95
Sex (female vs male)	2.37	0.57–9.84	0.23
Smoking history (no vs yes)	2.69	0.50–14.53	0.25
PS (0–1vs 2–3)	2.19	0.23–20.12	0.49
Histology (Ad vs non-Ad)	0.60	0.12–2.85	0.53
Hypertension (no vs yes)	0.78	0.22–2.69	0.69
Diabetes (no vs yes)	1.46	0.33–6.34	0.57
Hyperlipidemia (no vs yes)	0.31	0.37–6.37	0.29
OMI (no vs yes)	0.70	0.08–6.07	0.75
Atrial fibrillation (no vs yes)	1.70	0.18–15.41	0.69
Brain metastasis (no vs yes)	5.24	1.72–16.10	0.004

*PS* performance status, *Ad* adenocarcinoma, *OMI* old myocardial infarction, *95 % CI* 95 % confidential interval

**Table 5:** Incidence of CI and fatality rate

	Total patients	BM (−)	BM (+)
Numbers	515	355	160
CI (+)	15	5	10
Incidence	2.9 %	1.4 %	6.3 %
Death	8	2	6
Fatality rate	53.3 %	40.0 %	60 %

*CI* cerebral infarction, *BM* brain metastasis

### Survival after CI and survival of patients with and without CI

**Fig. 2:**
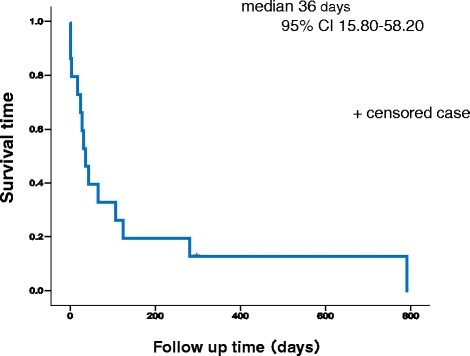
Survival time from development of cerebral infarction. Kaplan–Meier plot of survival time from diagnosis with cerebral infarction

**Fig. 3:**
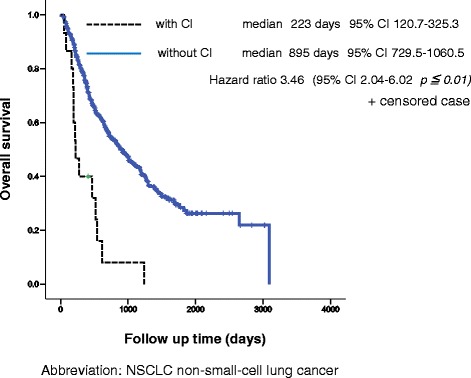
Overall survival from diagnosis of advanced or recurrent non-small cell lung cancer. Kaplan–Meier plot of overall survival for patients with cerebral infarction and without cerebral infarction

Of the 15 patients who developed CI after diagnosis of advanced or post-operative recurrent NSCLC, 8 patients (53.3 %) died of CI. The median survival time (MST) was 36 days, and 1-year survival rate was 6.7 % after development of CI (Fig. 2). OS from diagnosis of advanced NSCLC or post-operative recurrence was significantly shorter in patients with CI than in patients without CI (223 days versus 895 days; HR, 3.46; 95 % confidence interval, 2.04–6.02; *p =* 0.001*,* Fig. 3).

## Discussion

In this study, we found that the incidence of CI was 2.9 % in advanced or post-operative recurrent NSCLC, and brain metastasis was the only risk factor for the development of CI in patients with advanced or post-operative recurrent NSCLC. In addition, the incidence of CI in patients with brain metastasis was significantly higher than that in patients without brain metastasis.

Few published studies have evaluated the association between CI and advanced NSCLC. Population-based data from Taiwan National Health Insurance revealed an increased risk of CI in lung cancer patients [4]. In this report, the incidence of CI in all the stages of lung cancer was 21.80 per 1000 person-years. Conversely, the Dutch Pathology Registry linked to the PHARMO (Pharmaco Morbidity) medical record linkage system did not reveal increased risk of CI in lung cancer patients [5]. In this report, the incidence of CI in all stages and histological types of lung cancer was 3.8 per 1000 person-years in 6 months after diagnosis with lung cancer. However, the incidence of CI in patients with advanced or post-operative recurrent NSCLC has not been reported yet. Two retrospective studies investigated the incidence of and risk factors for thromboembolic events including CI in lung cancer patients [6, 7]. However, these reports included all stages and histological types of lung cancer and did not focus on CI. The current study is the first to evaluate the incidence of CI in advanced NSCLC, and the risk factors for CI in advanced NSCLC in terms of patient characteristics and complications.

An association between malignant neoplasms and thromboembolism, including arterial thromboembolic events and venous thromboembolism (VTE), has been reported by several groups. It has been reported that 13.2 % of newly diagnosed NSCLC patients have VTE [8]. In that report, an increase in white blood cells was associated with VTE. Another group reported that incidence of VTE was higher among lung cancer patients with adenocarcinoma histology than among those with squamous cell carcinoma histology [9]. Thromboembolism may be associated with poorly differentiated adenocarcinoma. Few case reports have described an association between poorly differentiated adenocarcinoma and thromboembolism in pancreatic, breast, and advanced ovarian cancers [10–12]. In our study, 12 patients with CI had adenocarcinoma. However, there were no significant differences in histology between patients with CI and without CI. Further investigations with larger study populations are warranted to determine whether poorly differentiated adenocarcinoma is a risk factor for CI.

In general, CI was categorized as cardiac embolism, atherosclerosis, and lacunar infarction. On the other hand, it is difficult to easily classify etiology of CI in these patients. However, all of the patients with CI in our study had a sudden onset of severe neurological symptoms such as paralysis, dysarthria, and disturbance of consciousness. Therefore, lacunar infarction could not be an etiology of the CI in these patients. In our study, 10 patients had multiple infarctions and 5 patients had a single infarction on brain MRI. In patients with multiple infarctions, the CI must have been caused by thromboembolism. Seven patients had hypertension, and 1 patient had hyperlipidemia. In these patients, the occurrence of atherosclerosis might be related to the CI, although the development of thromboembolism/coagulation disorder is also related to CI. Some papers discuss the causes of CI in patients with cancer. One report described five causes of CI: direct tumor relation, coagulation disorders, infection, treatment related and paraneoplastic [13]. In terms of coagulation disorders, cancer is known as one of the acquired prothrombotic states related to a high risk for stroke [14]. Disseminated intravascular coagulation (DIC) is usually associated with advanced cancer. In this case, levels of specific markers for DIC, including d-dimer, are often elevated. It has been reported that platinum compounds are associated with a higher risk for stroke including CI [15]. Circulating endothelial-derived and platelet-derived microparticles occurring during the third or fourth infusion might contribute to cisplatin-induced stroke [16]. Other anticancer agents, including cyclophosphamide, 5-fluorouracil, paclitaxel, and methotrexate, have been associated with the development of CI [13, 17, 18]. Whereas 5 patients received chemotherapy with platinum agent (CDDP + PEM, CBDCA + PEM, and CBDCA + PTX), other patients were treated with PEM, DTX, EGFR-TKI or crizotinib at the onset of CI. Therefore, it is unclear that incidence of CI in patients treated with platinum agent is higher than in patients received non-platinum agent in this research. Most of the patients developed CI during first or second line chemotherapy. Because CI tends to occur immediately after diagnosis with advanced or post-operative recurrent NSCLC, attention should be paid if the patients have the risk of CI.

Ten patients had multiple micro infarctions, and 5 had single large infarctions on brain MRI. There was no evidence of direct relation of tumor on brain MRI. However, taking into account the fact that the incidence of CI in patients with brain metastasis was significantly higher than that in patients without brain metastasis, invasion or tumor emboli to vessels might cause CI. Moreover, cancer tissue releases the accelerator of coagulation and cause prothrombotic states. When the patients have brain metastases, accelerator of coagulation will increase locally and elevate the risk of CI. To our knowledge, there has been no report on the association between brain metastasis from any cancer and CI. However, it has been reported that the incidence ratio for subsequent ischemic stroke within 6 months after diagnosis in patients with cancer of the nervous system is the highest among all patients with any type of cancer [19]. This report might support the findings of our study. There were no cardiogenic embolisms by physical examination and echocardiography, and the cause of CI in 10 patients with multiple and micro CIs could be categorized as coagulation disorders. CI developed during treatment in 14 out of 15 patients, and the cause of these CI could be categorized as treatment related. Of these patients, 12 developed CI during chemotherapy. Cytotoxic agents have been reported as risk factors for CI; however, 4 patients in our study experienced CI during treatment with molecular targeted agents.

Only one patient with CI was able to receive anticancer therapy after development of CI. Median survival time from onset of CI was 36 days. OS in patients with CI was significantly shorter than that in those without CI. These findings suggest that development of CI contributed to a worse prognosis in patients with advanced and recurrent NSCLC. Generally, the prognosis of advanced NSCLC patients is improving due to the development of chemotherapy, and control of adverse events and complications during chemotherapy, including CI, is becoming more important. The prophylaxis of CI in advanced NSCLC during treatment should be examined in future studies.

This analysis has several limitations. First, this was a retrospective study. However, although we might miss asymptomatic CI, the onset of CI is easy to detect, and therefore its frequency might not differ significantly from prospective evaluations. Moreover, asymptomatic CI is not significant in clinical practice. Secondly, this study did not include blood test findings in the analysis of risk factors for CI. However, although a study revealed an increased white blood cell count associated with VTE, blood test findings are not commonly known as risk factors for CI, and we assessed hyperlipidemia as a risk factor.

## Conclusions

This is the first study to evaluate the incidence of CI in advanced or post-operative recurrent NSCLC and the risk factors for CI in advanced or post-operative recurrent NSCLC in terms of patient characteristics and complications. Our findings suggest that the incidence of CI is high in advanced or post-operative recurrent NSCLC, and especially higher in patients with brain metastasis than in those without brain metastasis. The prophylaxis of CI in advanced NSCLC during treatment should be considered in future studies.

## Abbreviations

CBDCA: carboplatin
CDDP: cisplatin
CI: cerebral infarction
DIC: disseminated intravascular coagulation
ECOG: Eastern Cooperative Oncology Group
EGFR: epidermal growth factor receptor
HR: hazard ratio
MRI: magnetic resonance imaging
MST: median survival time
NSCLC: non-small cell lung cancer
OR: odds rate
OS: overall survival
PEM: pemetrexed
PS: performance status
PTX: paclitaxel
VTE: venous thromboembolism
